# Imitation by social interaction? Analysis of a minimal agent-based model of the correspondence problem

**DOI:** 10.3389/fnhum.2012.00202

**Published:** 2012-07-04

**Authors:** Tom Froese, Charles Lenay, Takashi Ikegami

**Affiliations:** ^1^Ikegami Laboratory, Department of General Systems Studies, Graduate School of Arts and Sciences, University of TokyoTokyo, Japan; ^2^COSTECH, University of Technology of CompiegneFrance

**Keywords:** social cognition, interaction studies, evolutionary robotics, dynamical systems theory

## Abstract

One of the major challenges faced by explanations of imitation is the “correspondence problem”: how is an agent able to match its bodily expression to the observed bodily expression of another agent, especially when there is no possibility of external self-observation? Current theories only consider the possibility of an innate or acquired matching mechanism belonging to an isolated individual. In this paper we evaluate an alternative that situates the explanation of imitation in the inter-individual dynamics of the interaction process itself. We implemented a minimal model of two interacting agents based on a recent psychological study of imitative behavior during minimalist perceptual crossing. The agents cannot sense the configuration of their own body, and do not have access to other's body configuration, either. And yet surprisingly they are still capable of converging on matching bodily configurations. Analysis revealed that the agents solved this version of the correspondence problem in terms of collective properties of the interaction process. Contrary to the assumption that such properties merely serve as external input or scaffolding for individual mechanisms, it was found that the behavioral dynamics were distributed across the model as a whole.

## Introduction

The study of imitative behavior is a central topic in developmental and comparative psychology, as well as in social neuroscience (Heyes, 2009). It is widely accepted that imitation plays a significant role in human social learning and enculturation, and that it serves as a cultural inheritance mechanism for human-specific cumulative cultural evolution (Tomasello, 2001). Imitation is a broad concept, but in current research it is often narrowly defined as the intentional behavioral matching of the precise means of a perceived action in order to achieve the same end.

One important challenge for a neuroscientific theory of imitation is to account for what is known as the “perceptual-motor translation problem” (Heyes, 2001) or the “correspondence problem” (Brass and Heyes, 2005): how can one agent's perception of another agent's behavior constrain its own internal motor system so as to produce a imitative behavior? The difficulty of such behavioral matching partly derives from the fact that we cannot directly perceive the other's internal motor configuration but only their behavioral expression. We can perceive *that* a person is performing an action, for instance that she is wiggling her ears, but not *how* that action is initiated.

In addition, in some situations we cannot perceive the bodily expression of our own actions, and so it is impossible to employ self-observation in order to monitor whether a match has been achieved (e.g., imitation of a facial expression). We normally do not worry about this fact. As adults we have already accumulated an extensive repertoire of implicit bodily know-how, i.e., a “body schema,” and we have acquired an intuitive appreciation of how our body is perceived externally, i.e., a “body image” (Gallagher, 2005). However, neonates have never seen their own face, and have little experience of other faces, so how can they imitate arbitrary facial gestures that are unlikely to be innate reflexes? We will refer to this particular problem of neonatal facial imitation as the “strong correspondence problem.” Meltzoff and Decety (2003) have called this the “holy grail” of imitation research.

Infants can see the adult's face but can not see their own faces. They can feel their own faces move, but have no access to the feelings of movement in the other. If they are young enough they will have never seen their own face. There are no mirrors in the womb. The holy grail for cognitive- and neuro-science theories of imitation is to elucidate the mechanism by which infants connect the felt but unseen movements of the self with the seen but unfelt movements of the other. (Meltzoff and Decety, 2003, 491).

Heyes and Bird (2007) categorize solutions to the correspondence problem along two dimensions regarding (1) the origins of the mechanism, and (2) the functioning of the mechanism. In terms of (1) the central debate is about the role of evolution by natural selection versus lifetime learning, and in terms of (2) the main question is whether the imitation mechanism is primarily based on lower-level sensorimotor embodiment or if it also requires “higher-level” conceptual mediation. During the cognitivist revolution in the 1970s, one popular theory of imitation proposed an innate mechanism that is representationally mediated by higher-level cognition. This proposal was inspired by prominent evidence that human neonates can spontaneously imitate different arbitrary facial gestures (Meltzoff and Moore, 1977). Although versions of this kind of “innate” and “top-down” theory continue to persist in the literature (e.g., Meltzoff and Moore, 1997; Csibra, 2007) they are now in the minority. This change in outlook was prompted by a reassessment of the evidence for neonatal imitation (Jones, 2009; Ray and Heyes, 2011), and especially by the influential discovery of “mirror neurons” (Gallese et al., 1996). Thus, recent theories emphasize “lower-level” sensorimotor neural mechanisms, and they also appeal to the essential role of lifetime modifications of neural organization resulting from learning (e.g., Keysers and Perrett, 2004; Heyes, 2005; Rizzolatti, 2005; Hurley, 2008).

### Toward an interactive theory of imitation

We agree with the general trend of these developments. However, we suggest that the debate about imitation could further benefit from considering the role of interaction in meaningful social contexts as another relevant explanatory factor. In other words, we propose to expand the analysis of Heyes and Bird (2007) with another dimension along which to categorize theories of imitation, namely the *location* of the mechanism underlying imitation. In contrast to the prevalent internalist theories, there is also the possibility of a relational theory of imitation that is focused on the constitutive role of social interaction (Lenay and Stewart, 2012). So far relational approaches have not received much attention in the general debate about social cognition.

Most of social neuroscience has proceeded under the assumption that an isolated individual is the sufficient explanatory unit of analysis to account for social cognition, thereby focusing on their “social brain” (Frith and Frith, 2010). This assumption is sometimes referred to as methodological individualism (Boden, 2006). One important reason for the popularity of this internalist approach is that an isolated brain is much easier to study. Activity that is internal to the individual agent can be better located, measured, and made visible through imaging technologies, and this kind of social neuroscience has indeed been highly successful (Adolphs, 2010). However, there is also increasing interest in establishing a “second-person neuroscience” (Schilbach et al., forthcoming). One motivation for this change in perspective is the idea that the neural processes that are constitutive of detached and passive social observation may be different from the neural processes that are constitutive of immediate and active social engagement with others. Another motivation is the idea that the latter processes may not be limited to one individual alone; perhaps they derive from, and are maybe even constituted by, social interaction with other individuals^1^.

However, relational accounts of social cognition are confronted by conceptual and methodological challenges because it is difficult to capture social engagement “in the act.” Nevertheless, progress has been made in the study of interaction dynamics, especially by keeping the complexity of the social situation to an absolute minimum (e.g., Auvray et al., 2009; Lenay et al., 2011). This reduction of complexity has been aided by the design of minimal human-computer interfaces with the goal of enabling the systematic study of behavior under highly controlled conditions. We highlight two advantages of this method. First, much of the exploratory activity of perception is usually hidden from view, but by mediating this activity through a suitable interface it can be made visible in terms of the participant's behavior (Lenay and Steiner, 2010). Second, by reducing the range of possible actions and sensations to an absolute minimum, we can investigate what are the necessary and sufficient conditions of a behavior.

This kind of minimalist approach has been applied to the case of mimicry of bodily expression by Lenay and Stewart (2012; this issue, experiment 3). Briefly, in this study of “mimetic dynamics in the perceptual crossing,” two adult participants interact with each other in a 1D virtual environment via custom-built human-computer interfaces in order to mimic the bodily configurations of their virtual avatars, although by design they cannot know the specific configurations. Despite this extreme poverty of the stimulus, participants were successful at matching their configurations. An analysis of the results revealed that participants were sensitive to the collective properties of the interaction process, and adapted their bodily configuration accordingly. This supports an interactive account of solving the strong correspondence problem.

The significance of these findings for the debate about current theories of imitation is that social situations, which to an external observer exhibit forms of mimicry, do not necessarily require the postulation of individual (innate or acquired) mechanisms and intentions for imitation. This is because the experiment has shown that mimicry can also be an emergent outcome of certain kinds of social interaction. According to this interactive account of imitation, it is conceivable that a sense of mutual agreement in interaction grounds and precedes an explicit awareness about the bodily basis for that agreement (i.e., social understanding is primary, reflection about the fact that there is a matching of bodily expressions is secondary). Lenay and Stewart therefore propose that the classical logic of neonatal imitation could be inverted: mimicry spontaneously results from the mutual regulation of collective interaction dynamics, and it is this social interaction which provides the newborn with the motivation and means for linking her perception of the other with her proprioceptive sensations. It is only later in development that the child will discover that what she is doing during these situations is in fact an imitation or a matching of bodily expressions.

Not everyone will be convinced by the findings of this experiment. We highlight three potential concerns. It could be argued that the experiment has no direct implications for neonatal facial imitation (or imitation among non-human primates), because (1) it has not yet been demonstrated that the result is generalizable beyond the conditions of the experimental setup. More specifically, (2) it is possible that task success depends on sophisticated cognitive capacities that are only available to enculturated adult human beings. And (3) even if it is conceded that some collective properties of the interaction process play a role in shaping the solution, it could still be claimed that these properties only serve as additional input or external “scaffolding” for cognitive mechanisms that are ultimately isolated within an individual agent (e.g., Herschbach, 2012; Michael and Overgaard, 2012).

### Modeling social interaction

In order to respond to these potential criticisms, we applied an evolutionary robotics approach to Lenay and Stewart's (2012) experimental setup. In brief, evolutionary robotics is a synthetic method in which the experimenter designs and implements a task-environment of interest, specifies the embodiment of one or more robotic agents, and formulates a procedure for evaluating behavioral success (Harvey et al., 2005). The neural controller of an agent is simply a generic dynamical system, which is then optimized automatically according to the evaluation function, typically by means of an evolutionary algorithm. Although some researchers prefer to use physical robots, much work is based on computer models of “minimal cognition” (e.g., Beer, 2003).

There are several advantages to using this method. In contrast to actual psychological experiments and realistic neural models, the experimenter can reduce the complexity to a bare minimum in order to enable a holistic understanding of the mechanisms underlying the behavior. All parameters and variables of the brain-body-environment system are measurable and controllable, which allows a detailed and systematic study of how behavior emerges out of the interplay between various subsystems. In contrast to the fully pre-designed systems familiar from traditional AI, the experimenter is prevented from overly biasing the realization of the behavioral mechanism, which is instead the outcome of an opportunistic evolutionary process.

This synthetic method has been used to show that in some cases the mechanisms of social interaction can be distributed across two or more agents (e.g., Di Paolo, 2000; Quinn et al., 2003). Some studies are directly inspired by psychological experiments, for example Murray and Trevarthen (1985) “double video” paradigm (e.g., Ikegami and Iizuka, 2007; Di Paolo et al., 2008; Froese and Di Paolo, 2008). In particular, minimalist psychological experiments make suitable modeling targets (e.g., Di Paolo et al., 2008; Froese and Di Paolo, 2010, 2011). Of course, these results do not have the same status as empirical data, but they function as intuition pumps and thought experiments (Di Paolo et al., 2000). They help us to refine existing theories, provide proof of concepts, and generate new insights that can lead to further psychological experiments (Rohde, 2010).

By using evolutionary robotics to implement a model of Lenay and Stewart's study of mimicry, we respond to the potential criticisms as follows. Regarding (1) the problem of generalization we show that the essential results of the psychological experiment can be replicated in a different medium, in this case a minimal dynamical system. This also mitigates (2) the worry about requiring sophisticated cognition, because the simulated agents are governed by “brains” that are far too minimal to contain any sophisticated cognitive mechanisms. Therefore, any real brain will have sufficient complexity to realize the dynamics found in the artificial neural network. In response to (3) the possibility of explanations based on methodological individualism, we clarify the relationship between the internal dynamics, individual behavior, and the interaction process as a whole. We show that these components cannot be clearly separated. More generally, the analysis of the model sheds new light on the interpretation of the empirical data, and it allows us to propose new hypotheses that can be tested by further psychological experiments.

## Methods

### A minimal psychological experiment of imitation

**Figure 1:**
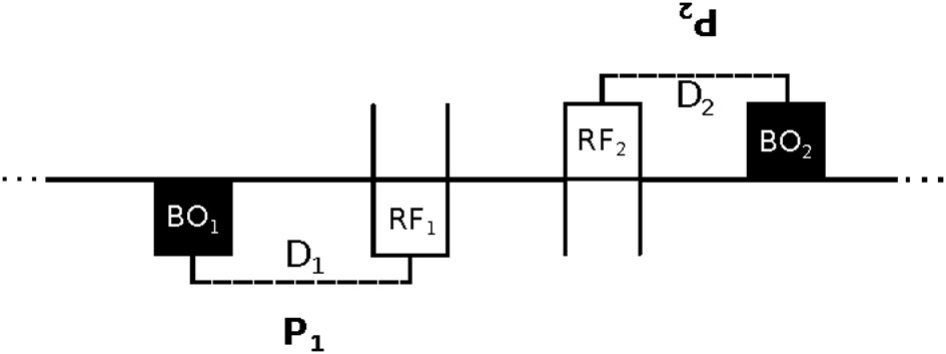
**Schematic of the experimental setup.** Two participants (P_1_ and P_2_) face each other in a 1D virtual environment (horizontal line). Participants cannot see the overall status of the environment. By using a computer mouse they can move their receptor field (RF) to detect the presence of the other's “body-object” (BO). They only receive tactile feedback as long as their RF overlaps the other's BO. The movement of a participant's BO and RF are connected by a rigid link. Link distance D is measured as the relative position of a BO in relation to its RF (in this case D_1_ < 0 and D_2_ > 0). At the start of a trial each D is initialized to a different length that is unknown to the participants. Participants can adjust their D by shifting the BO's egocentric position left- and right-wards by left- and right-clicking, respectively. As shown in the figure, a situation of “mimicry” occurs when participants have complementary configurations (i.e., D_1_ = -D_2_). Since participants are unaware of their own bodily configuration and that of the other, achieving this mimicry models the essential elements of the strong correspondence problem of neonatal facial imitation.

In order to evaluate the possibility of an interactive explanation of imitation, Lenay and Stewart (2012) created a modified version of the minimal technological setup that was used by Auvray et al. (2009) for a related psychological study of social interaction. Lenay and Stewart tried to recreate the essential elements of the strong correspondence problem of neonatal facial imitation for adult participants in a minimal virtual environment. They designed a new human-computer interface through which two adult human participants can explore a 1D circular virtual space and interact with each other. The interface consists of a tactile feedback device, which provides an all-or-nothing stimulus to a participant's finger, and a computer mouse by which participants can alter their position in the virtual space. Both participants are represented in the virtual space in a twofold manner, namely as a “body-object” (BO) and as a “receptor field” (RF). Loosely speaking, the BO represents a participant's body as the other perceives it, and the RF represents a participant's subjective gaze, which the other cannot directly perceive. All RFs and BOs have the same length. The experimental setup is shown schematically in Figure 1.

The movements of a participant's BO and RF are linked by a rigid connection, which is initialized at the start of a trial to a random relative distance that is unknown to the participants. This distance is referred to as D_1_ for participant P_1_ and D_2_ for the other participant P_2_; its algebraic value is negative if a BO is to the left of its RF, and positive if it is on the right-hand side (calculated from allocentric coordinates). Direct knowledge of the situation is reduced to an absolute minimum: a participant only receives tactile stimulation as long as her RF overlaps the BO of the other participant, and no feedback otherwise. Accordingly, this minimalist setup takes the traditional “poverty of the stimulus argument” (Ray and Heyes, 2011), which had been employed to argue for an innate mechanism, to the extreme. Traditionally, it was widely held that neonatal imitation could not be based on learning or interaction, because there is insufficient time and information for the infants to acquire the relevant expertise. The current setup takes an even stricter minimalism as its starting point, while at the same time also excluding the possibility of accounting for behavioral success in terms of specialized genetic factors (since we did not evolve to match virtual avatars).

Participants are capable of two kinds of action. They can use the computer mouse to move their virtual body position left and right along the 1D virtual environment. And they can also click the left and right button of the mouse to shift the position of their BO leftward and rightward, respectively (from an egocentric perspective around their RF). The task given to each participant is to locate the other in the virtual space, and to interact with the other by moving back and forth. They are also told that if they sense that the interaction process exhibits a general tendency to drift to the right they should click on the left-button, and vice versa. This is because sideways drift is an indication of a mismatch between their bodily configurations.

For instance, in the case of D_1_ + D_2_ < 0, if the RF_1_ overlaps the BO_2_, P_2_ will have to move to its right to find BO_1_; but then P_1_ will have to move to its left to recover BO_2_ once again, and so on, resulting in a collective drift of both participants in the same allocentric direction. From their egocentric perspective, P_1_ will experience this drift as going leftward, and P_2_ will experience it in a rightward direction, so P_1_ will tend to right-click and P_2_ will tend to left-click. Accordingly, they combine their efforts at reducing the relative difference between their bodily configurations.

This experimental setup may seem to be so minimal and artificial that it is difficult to relate it to the strong correspondence problem. However, it is a virtue of this kind of approach that the minimalist sensorimotor interface forces the perceptual activity of the participants to become visible in their interactions, thereby enabling a detailed study of their dynamics (Lenay and Steiner, 2010). The artificial setting also allows explicit control over various features of the situation. In particular, participants have no access to either of the two bodily configurations (i.e., neither D_1_ nor D_2_). It follows that in this study the emergence of mimicry cannot be explained by (1) intra-modal mapping, i.e., comparing external perception of the other's body configuration with external self-monitoring of one's own body configuration, nor by (2) inter-modal mapping, i.e., by comparing external perception of the other's body configuration with internal self-observation (Meltzoff and Moore, 1997). Both innate and acquired inter-modal mapping is excluded by design. The important point is that if participants can still manage to achieve a situation of mimicry (i.e., D_1_ = −D_2_) under these restricted conditions, this result cannot be explained by any of the traditional accounts.

Given this experimental setup, it was found by Lenay and Stewart that participants are generally able to solve this version of the correspondence problem successfully. The results demonstrate that participants are able to interact so as to adjust their bodily configuration in a complementary manner. Their respective links are finally matched in relative distance, even though at no point do they explicitly know their own bodily configuration nor that of the other. Instead, they somehow managed to achieve this mutual mimicry on the basis of interacting with each other in a rhythmic, oscillatory fashion. Behavior was not always highly synchronized; in some cases there was role-taking whereby one participant took the lead in moving and/or clicking. Analysis of the experimental results indicated that participants succeed in matching their bodies by responding to the relative stability of the interaction process, because, as described above, mismatches in relative bodily configuration introduce systematic sideward drifts into the flow of the interaction. This drift cannot be reduced to actions of one of the participants; on the contrary, both participants are subjected to this drift, which emerges out of their interaction. Mimicry was therefore enabled by a collective property of the interaction process as a whole.

### A minimal modeling experiment of imitation

The essential features of Lenay and Stewart's psychological experiment are retained in the model. Two simulated agents interact via a 1D virtual space, in which they are each embodied as a RF that is rigidly linked with a BO. The only important difference to the original experiment is that two minimal artificial neural network controllers replace the two adult human participants. We briefly describe how the experimental setup was redesigned as a computer model to help interpretation of the results; further technical implementation details can be found in the Appendix.

We followed the evolutionary robotics approach proposed by Beer (2003) by using a continuous-time recurrent neural network (CTRNN). The change in internal activity of a CTRNN is described by the following state equation.

$$\begin{array}{c} \tau_{i}{\dot{S}}_{i}=-S_{i}+\sum_{j=1}^{N}w_{ji}\sigma \left(g_{j}\left(S_{j}+\theta_{j}\right)\right)+I_{i}\\ i=1,\ldots ,N\\ \sigma \left(x\right)=1/\left(1+e^{-x}\right) \end{array}$$

These equations describe the state changes of a continuous dynamical system that is roughly analogous to the operation of an actual neuronal network, where *s* is the state of each neuron, τ is its time constant, *w*_*ji*_ is the strength of the connection from the *j*th to the *i*th neuron, *g* is a gain, θ is a bias term, and σ (*x*) is the output of a neuron given its state, which is defined by the standard logistic activation function (range [0, 1]). The gains *g*_*i*_ are all set to a constant of 1 and therefore have no effect on the system.

We acknowledge that the CTRNN controller is not a realistic model of the brain, let alone of a whole person. In order to make this crucial difference explicit we continue referring to a human person of the psychological experiment as a participant (P), while referring to a simulated person of the model as an “agent” (A). For our purposes we do not require a more complex model. We selected this type of artificial neural network because it is a popular choice for evolutionary robotics (e.g., Beer, 2003; Harvey et al., 2005). An advantage of using a CTRNN is that its dynamical properties are well understood, at least for small network sizes. It is a simple but dynamically universal neural network, and we are using it as a generic continuous-time dynamical system to model the temporal structure of the agents' behavior. The CTRNNs of the two agents are set to be structurally identical (i.e., all parameters and topology are the same), because participants of the actual psychological experiment are assumed to be interchangeable. Potential differences in personality type are therefore not explicitly modeled, although the internal states of the agents will of course differ depending on their respective histories of interaction. Since A_1_ and A_2_ face each other across a 1D environment, the sign of agent A_2_'s changes in position and link distance are inverted (i.e., multiplied by −1).

Each agent's CTRNN consists of eight neurons (*N* = 8) that are fully interconnected including self-connections. The first neuron is a receptor neuron receiving binary input from the agent's RF. The input represents a participant's all-or-nothing tactile sensation (i.e., 0 = no contact; 1 = contact). The second neuron is an effector neuron that regulates the continuous movement of the whole body configuration. This models a participant's movements with the mouse. The third and fourth neurons are also effector neurons, which control the left- and right-button clicks, respectively. The four remaining neurons are interneurons without any dedicated function.

Modeling the stepwise adjustment of a participant's link distance by means of left- and right-clicks was tricky, because it required mapping the CTRNN neuron outputs from continuous dynamics to a discrete domain. We chose to model a mouse click by implementing a button activation threshold. If a button neuron's output (range [0, 1]) increases to more than or equal to 0.75, then its button is turned “on” and produces a “click.” The button is turned “off” when that neuron's output falls below 0.75. In this way an agent cannot adjust its link continuously, because the button has to be turned off before it can be turned back on. The reason for these choices is to facilitate a distinction between the timescales of movement and link adjustment, which should be faster and slower, respectively. We modeled the activities of the two buttons with two distinct neurons, rather than with two activation thresholds of one neuron, because we believed that this might facilitate the evolution of flexible behavior.

For our model we slightly modified the standard CTRNN equation by including some additional gain parameters. First, the input *I* to the receptor neuron is multiplied by a gain *r*_*i*_. This gain modulates the strength by which the internal dynamics of the neuron are perturbed by input. Second, the output σ (*x*) of every effector neuron is multiplied by a gain *e*_*i*_. These gains modify the magnitude of the output effects, namely the range of movement velocity and the step size of link adjustment. Third, the output σ (*x*) of the movement neuron is linearly mapped from range from [0, 1] to [−1, 1] before the gain *e*_*i*_ is applied. This linear mapping has the effect of letting the neuron control both leftward and rightward motion. By adding these parameters to the CTRNN equations, we effectively placed some aspects of the agent's embodiment under the influence of the automatic optimization procedure. A standard evolutionary algorithm optimized the parameters. The evaluation function measured how well the agents were able to interact and to match their bodily configurations. Each pair of agents was evaluated for 15 trials of 3000 time steps each with different initial conditions (for details, see the Appendix).

The precise setup of these trials differed slightly from the original psychological study to facilitate the evolutionary process. The 1D space was not joined into a circle, but was infinitely long in practice given the short duration of a trial. This modification excluded the possibility of optimizing an otherwise common initial strategy, which consisted in interacting by repeatedly going around the circle. The size of the RFs and BOs was set to 1 arbitrary unit of space. Before the start of a trial the initial positions of the RFs of A_1_ and A_2_ were set to 10 and −10 units of space, while the distances to their BOs were initialized to D_1_ = −20 and D_2_ = 20 units of space, respectively. D_1_ was then varied by a random number drawn from a uniform distribution (range [−1.5, 1.5]), and the initial position of A_1_'s RF was also varied by a random number drawn from uniform distribution (range [−1.5, 1.5]). This procedure ensured that the agents started each trial in a configuration that was relatively advantageous for establishing an interaction process, and yet they still had to work out how to match their bodily configurations without knowing their status.

## Results

In order to facilitate the analysis of the modeling results we set the range of random variation that was normally applied to the initial position of RF_1_ to 0. Although the agents had been evolved to deal with the additional ambiguity of initial differences in position, here we are only interested in their ability to reduce differences in relative body configuration. We systematically varied distance D_1_ in the range [−1.5, 1.5] with an increment of 0.5, thereby producing data for 7 representative trials.

### Overview of the results

**Figure 2:**
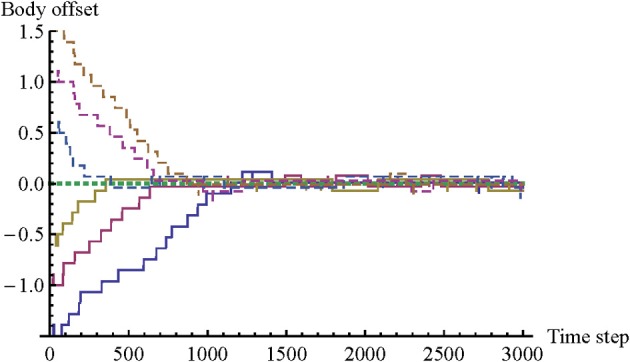
**Changes in “body offset” for seven illustrative trials.** We define “body offset” as the relative difference between body configurations (D_1_ + D_2_). A body offset of 0 is perfect mimicry. The graph demonstrates that the model qualitatively replicates two findings of the original psychological study. The agents are capable of mimicking each other's body configuration even without knowledge of their status, and there is a tendency for body offset to decrease all the way to 0, although mutual contact can already be made when body offset falls in the range [−1, 1].

We define “body offset” as the relative difference between body configurations, which can be calculated summing the link distances (D_1_ + D_2_). Body offset is an indication of mutual mimicry. An offset of 0 is a perfect match. As long as it is within the range [−1, 1] the agents can make contact with each other simultaneously. Typical changes in body offset are shown in Figure 2.

The changes in body offset demonstrate that the model has successfully replicated the main result of the original psychological experiment by Lenay and Stewart. In trials initialized with a non-zero body offset, the agents quickly reduce that offset toward 0, and in the trial initialized with a body offset of 0, the agents retain that offset. In other words, even though the agents do not know each other's body configurations, they are capable of mimicking each other's body configuration effectively.

There is another correspondence between the results of the model and the results of the original psychological study. In principle, participants could have stopped as soon as the body offset was close to 0; this would have entailed a perfect score without any need for further interaction or adjustment. However, Lenay and Stewart observed that in practice most participants continue to interact and to adjust link distances for the rest of the trial, while keeping body offset close to 0. Diversity of link distances tends to increase over time. One explanation for this trend is that the solution for the task belongs to an infinite class of situations where D_1_ + D_2_ = 0. But there must also be a motivation to continue interacting and clicking. Participants may become entrained in an interaction process that is to some extent self-sustaining. We can observe a similar kind of behavior in the current model, as show in Figure 4.

**Figure 3:**
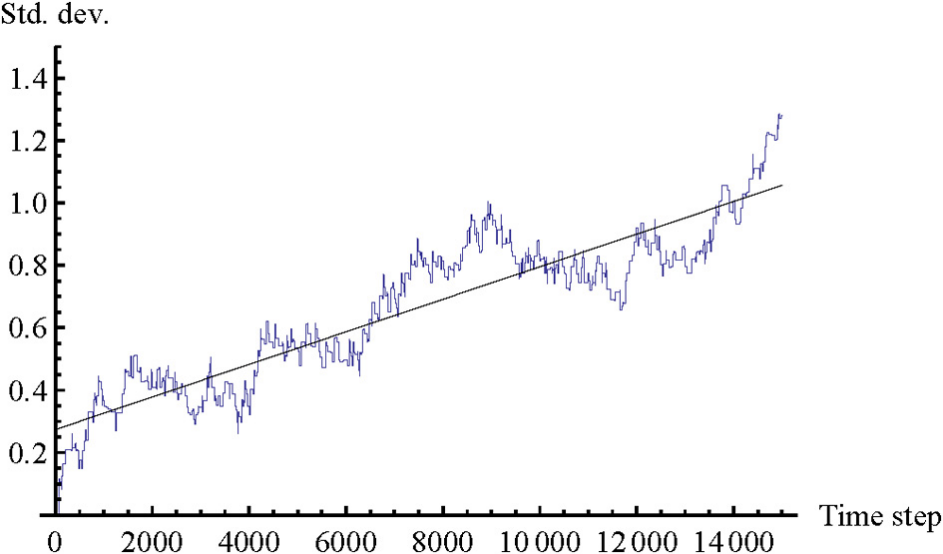
**Standard deviation of combined link distance (D_1_ −D_2_) in seven trials with identical initial conditions (initial body offset = 0).** Trials were run for an extended period of 15000 time steps to evaluate the long-term trend. Values of D_1_ and D_2_ were combined into one trajectory because the difference in standard deviation between D_1_ and D_2_ is negligible. The trend line represents a “best fit” linear regression. The graph demonstrates that the model qualitatively replicates another finding of the original psychological experiment: the diversity of link distances tends to increase throughout the whole trial, even after agents establish mutual perceptual crossing, and also when agents are initially set to perfectly mimic each other's body configurations.

Similar to the original psychological experiment, in the model we also find that the diversity of values of D_1_ and D_2_ continues to increase over the whole trial. After the agents have succeeded in reducing the body offset close to 0, which typically happens around time step 1000 already, they continue to adjust their link distances for the rest of the trial in a coordinated fashion. This behavior occurs even if agents start the trial with perfect mimicry (initial body offset = 0), as shown in Figure 3. Since this trend is observed even though the trials are started from identical initial conditions, including the states of the CTRNN neurons (which are always set to 0), the increase in diversity must be related to slight differences in the Gaussian noise applied to the movement neuron (see Appendix). Over time this noise will affect paths of motion, and therefore times of contact and interaction history. Fluctuations in movement can lead to onset and absence of contact when it is not expected, and therefore may produce an illusion of slight mismatch. Given these modeling insights, we hypothesize that the increase in link diversity in the psychological study can also be partially explained by the fact that participants do not have perfect control over their movements (e.g., due to various delays, inertia of arm motion, and inaccurate position measurement because of mouse skipping during fast movement).

**Figure 4:**
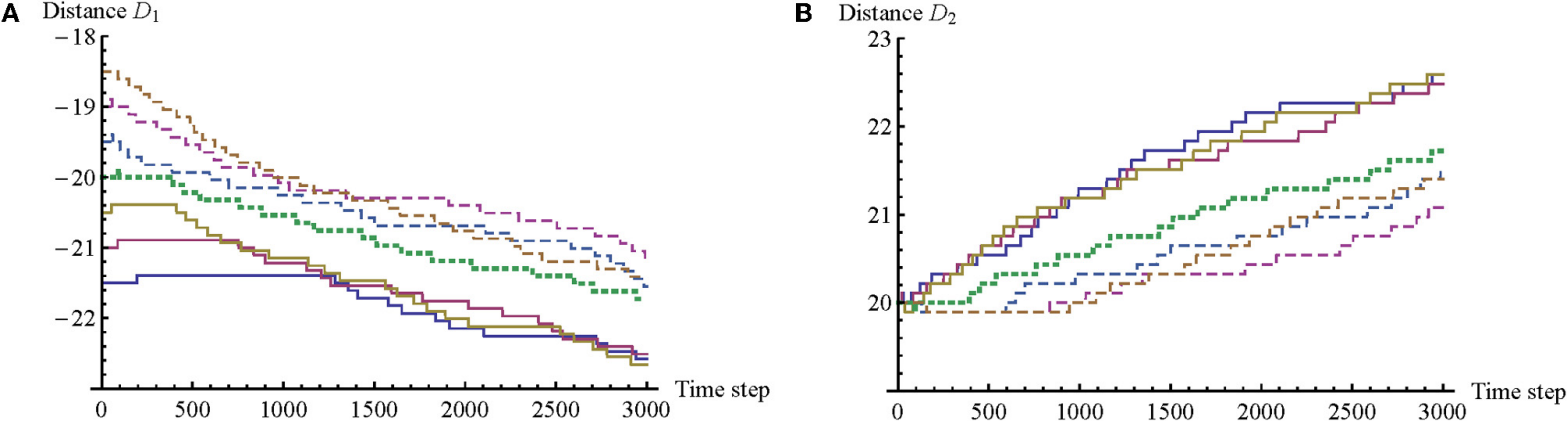
**Graphs of link distance D for seven illustrative trials.** Distance D measures the distance of an agent's BO from its RF along the 1D environment. **(A)** Changes in D_1_. There is a correlation between positive values of body offset, e.g., larger initial values of D_1_ (dashed lines), and shorter times for A_1_ to begin regular adjustments of D_1_. **(B)** Changes in D_2_. Here the opposite trend can be observed; there is a tendency for A_2_ to start modifying D_2_ sooner for negative values of body offset, e.g., smaller initial values of D_1_ (solid lines). A similar kind of differentiation between clicking and non-clicking roles was found in the psychological experiment.

There is another correspondence with the findings of the original psychological study. Lenay and Stewart report that in all cases participants were actively moving to obtain sensory stimulations, i.e., they were performing a kind of active perception. However, it was found that quite often, in one-third of all trials, only one participant engages in clicking behavior, thus changing either D_1_ or D_2_, while the other participant is only active in maintaining the interaction. This differentiation into distinct roles is possible because body offset is the sum of distances (D_1_ + D_2_) and can therefore be regulated by each participant alone. A similar differentiation between clicking and non-clicking roles was found in the model, as is shown in Figure 4.

In the model the assignment of these roles is related to body offset. When a trial starts with a negative body offset (e.g., −1.5), A_2_ begins with the clicking behavior, while A_1_ only begins clicking much later, sometime after the possibility for mutual perceptual crossing has already been established. When a trial starts with a positive body offset, the opposite differentiation of roles is observed. And in the case of no initial body offset (e.g., D_1_ = −20; D_2_ = 20), no clear differentiation into roles is observed. We note that this role division was first discovered in the model, and only subsequently did Lenay and Stewart confirm that role division indeed took place in one-third of their trials as well. We can derive further predictions about the empirical data that still need to be confirmed: (1) there is a correlation between size of body offset and likelihood of role division, such that (2) a larger initial offset indicates a greater likelihood of role division; and (3) there is a tendency for role division to disappear after the possibility of simultaneous perceptual crossing has been established. A preliminary review of the empirical data revealed that these predictions are only partially fulfilled; role-taking appears to be more complex in the case of human participants.

The graphs in Figure 4 reveal more trends. An agent tends to modify the distance of its link always in the same direction, and it retains this same direction across all of the trials. Agents also tend to always increase the absolute link distance. More precisely, it turns out that the agents have adopted a strategy that relies on making use of the left-button only. Although this behavior is unexpected, it is understandable because it decreases the complexity of the problem to be solved by a single agent as long as it is cooperating with the other agent. Now each agent only has to choose between two rather than four link-related actions, i.e., left-click or no left-click. And if the body offset happens to be such that an agent would have to right-click to correct it, then it simply waits for the other agent to left-click instead, because this amounts to the same overall change in body offset. In this way the solution to the task has been simplified via coordinated turn taking. On the basis of these findings we can derive additional predictions about the empirical data: (4) once participants start modifying the distance of their link, they tend to modify it in the same direction for the duration of a trial; and (5) participants do not make use of both buttons with equal probability. Again, on the basis of a preliminary analysis of the empirical data, it seems that human participants may not use both buttons equally, but they nevertheless tend to use both of them.

What the single-button solution demonstrates is how evolution will opportunistically select behavioral mechanisms that will “offload” task complexity into the interaction process, at least under stable social situations. We can therefore hypothesize that (6) a second button is not essential to the design of the experimental setup, although human participants tend to take advantage of it when it is provided.

### Analysis of a representative trial

**Figure 5:**
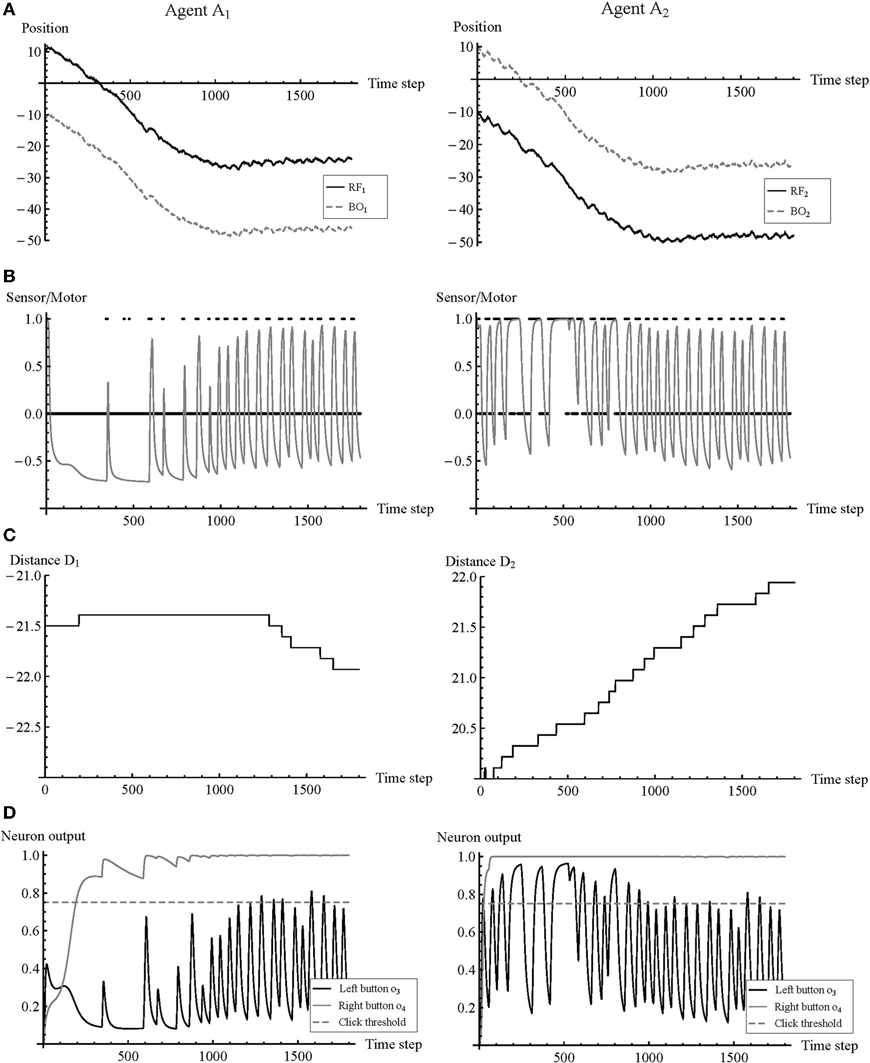
**Time series of the first 1800 time steps of a representative trial (initial body offset = −1.5).** Selected variables of agents A_1_ and A_2_ are shown in the left and right columns, respectively. **(A)** Position trajectories of RF and BO. **(B)** Input from contact sensor (black points) and mapped motor output (gray solid line) without gain, indicating the agent's movement. **(C)** Link distance D. **(D)** Output σ(*x*) of the effector neurons controlling left and right button pressings. A button is clicked whenever output crosses the threshold from less than to more than 0.75.

In order to better understand the strategy of the agents, we can analyze more closely the time series of a representative trial such as that shown in Figure 5. The trial is the same as that shown in Figure 2 where initial body offset is equal to −1.5.

This trial begins with the following situation. A_1_'s (RF_1_) and A_2_'s (BO_2_) both start from position 10, such that A_1_ receives input. However, A_2_'s (RF_2_) and A_1_'s (BO_1_) start from positions −10 and −11.5, respectively. Given that each object is only 1 unit of space wide, A_2_ will start without input. A_2_ begins searching in order to make contact with BO_1_ by moving RF_2_. But this movement pulls along BO_2_, thereby removing it from RF_1_. Accordingly, A_1_ no longer receives input and starts searching for BO_2_. A_2_ is able to find BO_1_, but it is forced to maintain this contact while compensating for a leftward drift. This drift results from A_1_'s searching for BO_2_ and A_2_'s maintaining contact with BO_1_. During this phase, A_1_ remains without contact and is making no changes to D_1_. At the same time, A_2_ often makes prolonged contact and frequently increases D_2_. Around time step 500 the body offset is reduced below 1 and there is possibility of mutual contact. The interaction is now characterized by regular perceptual crossing. Around time step 1000 the leftward drift is eliminated, as body offset is effectively 0. After this point A_1_ starts adjusting D_1_, and both agents continue adjusting D_1_ and D_2_ in a complementary manner.

The agents succeeded at their task. But how did they know-how to adjust their link distances appropriately? Lenay and Stewart found two correlations in the data of the psychological experiment to which participants might be sensitive: (i) a decrease in body offset is accompanied by an increase in the frequency of stimulation; and (ii) a decrease in body offset is accompanied by a decrease in drift. The same correlations can be observed in the case of the model.

However, Lenay and Stewart do not clarify the kind of mechanism by which these two correlations are supposed to be turned into an effective action. Do they serve as additional input to the explicit cognition of the participants, perhaps via integrating proprioception and tactile sensation? Or do they constitute contextual “scaffolding” that implicitly guides a participant's action? These accounts do not require that the methodological individualism of traditional cognitive science is rejected in favor of a relational view of cognition (Herschbach, 2012; Michael and Overgaard, 2012). An alternative possibility is to treat the behavior of each participant as a distributed, relational phenomenon that emerges out of the coupling of a brain, body, environment systemic whole. On this view, we can hypothesize that the interaction process itself partially constitutes the regulation of the appropriate behavior. Although it is difficult to evaluate these possibilities in the case of the psychological experiment, in the case of the model we fortunately have complete access to the activity of the minimal “brains” of the simulated agents.

### Analysis of the CTRNN controller

**Figure 6:**
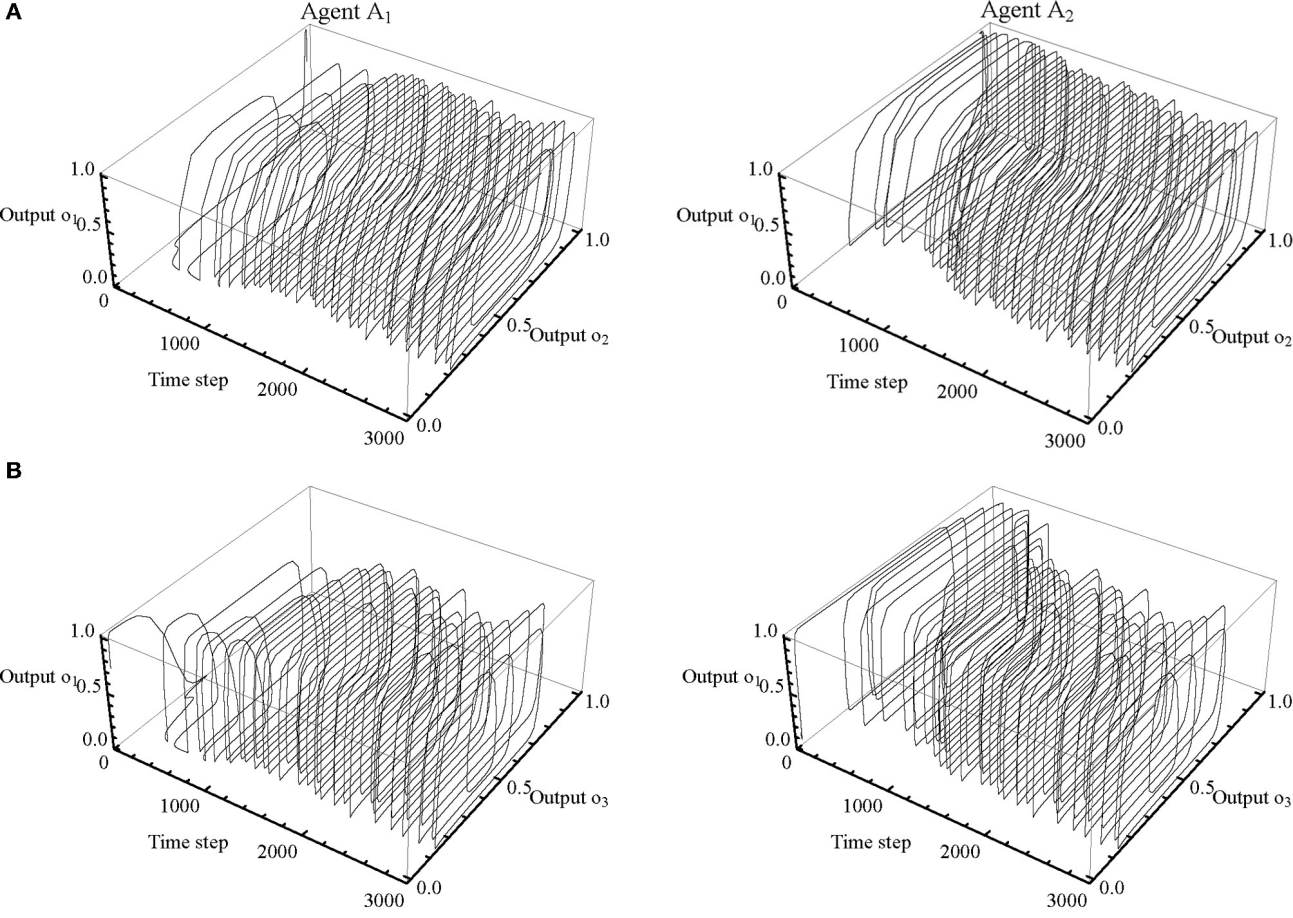
**Dependencies between receptor and effector neurons.** Subsections of the neural network's output trajectories for agents A_1_ (*left column*) and A_2_ (*right column*) shown for the first 700 time steps of the trial presented in Figure 5. **(A)** Relationship between the receptor neuron (*o*_1_) and the movement neuron (*o*_2_). Contact occurs when input to *o*_1_ is 1. Leftward movement occurs when *o*_2_ < 0.5; rightward movement occurs when *o*_2_ > 0.5. **(B)** Relationship between the receptor neuron (*o*_1_) and the left-button neuron (*o*_3_). A click is made every time *o*_3_ increases from less than to more than 0.75.

A preliminary analysis of the neural activity has shown that some neurons are largely redundant. It is therefore possible to simplify the analysis by focusing on a subset of neurons. Of particular interest is the relationship between the receptor neuron and the effector neurons, because this is how the agents internally regulate the sensory-motor loops that constitute their behavior. Moreover, the agents relied on a strategy that only required left-clicking to adjust the body offset. As can be seen in Figure 5D, output from the third effector neuron (*o*_4_) quickly saturates, and its role in the mechanism underlying behavior is therefore negligible. This allows us to further restrict the scope of the analysis to the “receptor” neuron (*y*_1_), the “movement” neuron (*y*_2_), and the “left-button” neuron (*y*_3_). Figure 6 shows how these three neurons are related in terms of their σ (*x*) output state space (*o*_1_, *o*_2_, and *o*_3_). We only show the first 700 time steps of the trial shown in Figure 5. The trajectories of A_2_ continue to be qualitatively similar after this point, while the trajectories of A_1_ will start to resemble the trajectories of A_2_, resulting in almost perfect symmetry by time step 1500.

The internal dynamics of A_1_'s sensory-motor loop are relatively straightforward at the beginning of this trial. After it looses contact with BO_2_, it continues searching in an attempt to regain contact. Most of the internal dynamics of A_2_ can be explained in terms of a transient cycle in 3D state space (defined by *o*_1_, *o*_2_, and *o*_3_). The timing of its receptor neuron's on/off switching is closely coupled with its oscillatory sideways movement. As long as input is present, A_2_ moves relatively quickly; when the input disappears because it moved to far ahead, A_2_ slows down until regaining contact, and so forth. This sensory-motor cycle regulates A_2_'s clicking as well. Prolonged contact causes the output of the left-button neuron to exceed the threshold of 0.75, which turns the button on and causes an adjustment of D_2_. Absence of contact allows output to decay below the threshold, thereby turning the button off again.

In order for this transient cycle in 3D state space to operate effectively, relative timing is of the essence. Duration of contact partially determines an agent's velocity, and is partially determined by it, because input stimulation increases velocity and therefore shortens duration. Duration of contact also partially determines an agent's clicking, because button activation requires input stimulation. Moreover, duration of contact is also an indicator of body offset. Since A_1_ will respond to stimulation with the same kind of oscillatory movement we described for A_2_ above, A_2_'s prolonged contact with BO_1_ must mean that RF_1_ has not yet made contact with BO_2_, i.e., there is no mutual contact between the agents. Accordingly, while A_1_ keeps searching without clicking, A_2_ extends D_2_ repeatedly by activating its left-button. This process continues until a situation of perceptual crossing is established. Once A_1_ begins to make contacts with sufficient duration, it will start adjusting D_1_ as well (after time step 1000).

This analysis has revealed another potential factor that could help to account for the performance of the participants in the psychological experiment, namely the duration of contact. Like frequency of contacts and magnitude of drift, duration is a property of the collective dynamics of the interaction process. However, an evolutionary robotics model of a related psychological study of perceptual crossing has shown that duration is not an essential aspect of that particular experimental setup (Froese and Di Paolo, 2011). Whether duration plays an essential role in the current setup can be addressed by future work, for example by setting object length to infinitely small points. For the present debate we are interested in the more general question of what role these kinds of properties of the collective dynamics could play in the generation of the behavior of the individual agents.

An analysis of the phase portrait of the CTRNN controller as a closed network reveals that a single globally attracting fixed-point governs its dynamics (data not shown). In the absence of any input, the network settles into this attractor after around 1500 time steps, and then remains static. From the perspective of methodological individualism, this lack of internal complexity should come as a surprise. How is it possible that such a single-attractor network processes the input sequence and regulates the output of the sensory-motor neurons appropriately? It turns out that the dynamics of the CTRNN during an interaction are partially constituted by the interaction process. The attractor shifts its position in state space as a function of the input parameter, thereby alternating the flow structure of the CTRNN state space between two distinct attractor configurations. The shifting pull of the attractor, combined with the non-linear constraints of the neural dynamics, regulates the internal activity and maintains it as a transient. In this way the structure and activity of the agent's “cognitive mechanism” is partially constituted in a relational manner [for a more detailed dynamical analysis of a similar finding, see Froese and Fuchs (2012)].

And it is not just the presence of contact that is important; absence of contact is just as essential. More precisely, it is the relative timing of the on/off status of the input that is constitutive of the appropriate regulation of the sensory-motor loop. Moreover, the timing of the on/off status of the contact sensor depends on the other agent's behavior as well; timing of contact is an emergent product of the behavior of the two agents as they interact. It follows that an agent cannot generate the required behavior without an appropriate process of interaction, such that an individual's behavior and the overall interaction process co-enable and co-determine each other. The strategy employed by the agents is inextricably distributed across the two agent system.

## Discussion

It remains to be seen whether a similarly distributed explanation of behavior can be provided in the case of the psychological experiment, and this largely depends on the analysis of the participants' internal dynamics. Some of these dynamics have already been externalized through the use of a minimal human-computer interface (Lenay and Steiner, 2010), but without a complementary way of understanding the role of brain activity, this is unlikely to convince hardnosed methodological individualists to give up internalist explanations just yet (e.g., Herschbach, 2012; Michael and Overgaard, 2012). Although there is increasing interest in the development of a second-person neuroscience (Schilbach et al., forthcoming), many formidable conceptual, methodological, and technical challenges still remain. Analyzing the phase portrait of a participant's nervous system is clearly out of the question, but there may be more easily detectable markers of a distributed cognitive process.

An advantage of the evolutionary robotics approach is that it helps us to clarify the conceptual possibilities on the basis of a more manageable minimal system, which is nevertheless able to qualitatively replicate essential aspects of the empirical data and can even predict new findings. The model can also serve as an intuition pump for the neuroscientific analysis of the psychological experiment. For instance, as can be seen in Figure 5, the frequent perceptual crossing between the agents is accompanied by a synchronization of their behavior. Starting around time step 1500, their neural activity becomes almost perfectly synchronized (Figure 5D). This is understandable given the essential part played by timing in the co-regulation of the internal dynamics of the agents' behavior. We therefore suggest that interactional and neural synchrony could play a similar role in the case of human participants, thereby extending the “binding-by-synchrony” hypothesis (Singer, 2007) to the case of social interaction. Dual-EEG recording during imitative social interaction has already provided evidence of inter-brain synchronization, although some asynchrony also appears to be important for differentiation of roles (Dumas et al., 2010, 2012). Interestingly, the model confirms this finding, because the disappearance of asynchrony coincides with the disappearance of the well-defined roles of “clicker” and “non-clicker.”

Although it is tempting to use this correspondence to further generalize the insights of the model to other kinds of social interaction, we have to proceed cautiously. Because the experimental setup requires that there is no familiarity with the other's bodily configuration or one's own, it is less applicable for explaining social interaction that permit direct observation of some kind. It is likely that there will still be a sense of pre-reflective bodily attunement during those situations, and that this experience can be explained in terms of the relative stability of the interaction process (Froese and Fuchs, 2012), but other important factors may also need to be taken into account.

More specifically, some concerns can be raised about how well the setup accounts for the situation of neonatal facial imitation. In terms of the model, it could be argued that such a distributed strategy only works because it has been “hardwired” by a process of evolutionary optimization onto a fixed experimental setup. The argument may correspondingly apply to the original psychological study. As a part of the experimental instructions, Lenay and Stewart explicitly told participants to click on the left-button if they felt the interaction drifting to the right, and vice versa. But is this information not simply solving the strong correspondence problem in advance? The worry is that both the psychological study and the model support the notion of imitation by interaction, but perhaps only on the basis of a pre-given source of knowledge about the situation, whether by oral instruction or genetic encoding. It could be argued that in order for the results of this setup to become more generalizable, it is important to investigate strategies that can succeed without this background knowledge.

We agree that the setup can be improved. As a first step, future work could randomly assign the function of the two buttons at the start of each trial, such that it is no longer immediately clear which of the buttons shifts the BO in which direction. In that case their functions have to be actively learned in some manner. A pilot study conducted by Lenay and Stewart has indicated that participants can still succeed under these conditions. We can understand this success because the basic nature of the solution to this correspondence problem remains the same: only a situation of mutual agreement enables a relatively stable interaction process, which means that appropriate actions can be relatively quickly learned by trial and error. No explicit verbal instruction (or genetic predisposition, as in the current model) is necessary to learn which actions improve the interaction. The crucial point is that this interactive solution to the strong correspondence problem places almost no demands on the individuals or the situation; this is what the minimal model has shown. We can therefore tentatively generalize the insights of the empirical and modeling studies: in some situations the emergence of mimicry during social interaction can be explained more parsimoniously by taking properties of the collective dynamics of the interaction process into account.

From the perspective of neonatal facial imitation, this insight could be understood as follows: an adult extends her tongue; the neonate starts moving her tongue, while at the same time closely observing the changing expression of the adult, until the point when there is an appropriate response of success from the adult. The actions of the adult person thereby serve as a kind of “mirror” for the neonate's own face. Regarding the possibility of this kind of interactive regulation, it is noteworthy that most studies in neonatal imitation have been explicitly designed so as to rule out the influence of interaction. Notably, Trevarthen (2005: 91–92) has complained about the inherent limitations of this kind of study: “By their nature, experiments in controlled laboratory situations must limit the subject's freedom to initiate communication inventively, or to test the consequences of their response. As a rule, Two-Way communication with the experimenter/observer is controlled out.” In other words, cognitive science has often explicitly prevented the possibility of mutual interaction playing any role, and thereby turned the internalist doctrine into a self-fulfilling hypothesis. The merit of the current experimental version of the correspondence problem is that it turns this convention on its head: methodological individualism is controlled out instead, and it is revealed that mimicry can still take place in terms of social interaction.

Interestingly, in their seminal paper on neonatal facial imitation, Meltzoff and Moore (1977, 76) acknowledge the difficulties of controlling the influence of interaction: “In reviewing films of the preliminary work, we also noticed that the examiner tended to alter the rhythm of his tongue protrusion as a function of the response of the infant.” Meltzoff and Moore regarded this rhythmic coordination as unwanted interference that had to be excluded in the design of the experiments. However, this is precisely the kind of interactive and temporally sensitive co-regulation of behavior that we also discovered by analyzing the model. Thus, even if the evidence for an innate and non-interactive mechanism of neonatal facial imitation is no longer compelling (Jones, 2009), there is still a promising possibility of an interactive account. We hypothesize that a neonate's ability to engage in flexible and consistent mimicry of arbitrary facial gestures constitutively depends on their engagement in meaningful social interaction.

### Conflict of interest statement

The authors declare that the research was conducted in the absence of any commercial or financial relationships that could be construed as a potential conflict of interest.

## Footnotes

^1^These ideas resonate with a number of other alternative traditions. In developmental psychology there is research of primary and secondary intersubjectivity (Reddy and Morris, 2004; Trevarthen, 2005). In philosophy of cognitive science there are phenomenological approaches to embodied intersubjectivity (e.g., Gallagher, 2001; Gallagher and Zahavi, 2008). There is the enactive approach to social cognition, which draws inspiration from a dynamical perspective, and which takes social interaction as its starting point (e.g., De Jaegher et al., 2010). These approaches are starting to form one coherent framework of research in social interaction (Froese and Gallagher, forthcoming).

## Appendix

### Technical details of the model

The CTRNN and the evolutionary algorithm were implemented on the basis of Beer's publicly available “Evolutionary Agents v1.1.2” C++ package^2^. The evolutionary algorithm was a simple genetic algorithm with rank-based selection; the maximum expected offspring of the highest ranked solution was set to 1.2. Due to the presence of a stochastic function in the evaluation stage (motor noise, see below), all solutions were evaluated again during each generation. The population consisted of 500 solutions. Each solution consisted of a sequence of genes, which were stored as real numbers and initialized with random numbers in the range [0, 1]. After each generation, the fittest 1% of the population was automatically copied into a new generation of 500 solutions. No recombination operator was used in the generation of offspring from selected parents. Instead, the genotype of the parent solution was copied, and a mutation operator changed the value of each gene with a small random value drawn from a Gaussian distribution with a variance of 0.1. If a mutation caused a gene to exceed this range, then the value was simply clipped at the boundaries ([0, 1]). In order to create a phenotype from a genetic solution, each gene was mapped to a parameter range of the CTRNN. The CTRNNs of the two agents are structurally identical. We made use of the following CTRNN parameter ranges: time constant τ range [1, 30], weight *w* range [−8, 8], bias θ range [−8, 8], receptor gain *r* range [−100, 100], and effector gain *e* range [0, 10].

Each trial was set to last 300 units of time, which were integrated using a Fourth-Order Runge-Kutta method with an integration step size of 0.1. The activations of all neurons were initialized to 0 before the start of the trial. At each time step a small random value drawn from a Gaussian distribution with a mean of 0 and a variance of 0.01 was added to the mapped output of the movement neuron before applying its effector gain. This addition of motor noise tends to enhance the robustness of the evolved solution. Trials are not initialized with identical seeds of the random number generator. In order to speed up the process of evaluation, trials were terminated early if an agents' RF was more than 20 units of space away from the other's BO. Each solution was evaluated for 15 trials. The scores of these 15 trials were weighted inversely proportional to their relative ranking, and then summed for the final score of that solution. Such rank-based weighting helps to prevent the evolutionary algorithm from optimizing some parameters at the expense of others.

Parameter optimization was divided into two main phases. First, the performance of a solution was measured in terms of the number of distinct contacts made during a trial. Note that this is not the same as rewarding simultaneous contact, which would require that the correspondence problem were already solved. We tried several other kinds of evaluation functions, but did not find them to be effective. An advantage of this initial evaluation criterion is that it favors the evolution of robust interaction, and it implicitly includes selection pressure for mimicry as well. This is because a relative difference of link distance, i.e., non-zero body offset (D_1_ + D_2_ ≠ 0), introduces a drift into the interaction process, and so the agents are forced to spend less time making contacts and more time chasing each other. In other words, agents that manage to eliminate the drift are able to interact more frequently. This phase was terminated as soon as one solution achieved over 100 contacts. This initial phase of evolution served an analogous function to the learning period of Lenay and Stewart's psychological study, during which participants were asked to familiarize themselves with the experimental setup and to maintain a stable interaction with each other.

For the second phase of evolution, the evaluation function was modified in order to explicitly measure the success of the agents at reducing body offset. Evaluation now consisted of two distinct components. First, we measured the extent of the agents to maintain close contact for as long as possible by dividing the total number of actual time steps (*t*_*total*_) by the maximum possible number of time steps (*t*_*max*_ = 3000). As before, a trial was terminated prematurely if an agent drifted more than 20 units of space apart from its target. Second, we measured the ability of the agents to match the relative distances of their BOs, i.e., to reduce the error of absolute body offset. We summed the error over all actual time steps, rather than just taking the final value, in order to encourage the agents to achieve mimicry as soon as possible after the start of a trial. Then we derived the maximum possible error by multiplying 1.5, which is the maximum magnitude of initial body offset, by the final number of time steps (*t*_*total*_). If the summed error was larger than the maximum error, for instance because the agents increased body offset beyond the pre-given initial variation (range [−1.5, 1.5]), then the summed error was set equal to the maximum error. The relationship between the summed and the maximum error is an indication of the success of the agents to reduce body offset. The combined evaluation function for the second stage was as follows:

$$trial\;score=\frac{t_{total}}{t_{\max}}+\frac{\left(1.5*t_{total}\right)-\sum_{t=0}^{t_{total}}abs{\left(D_{1}+D_{2}\right)}_{t}}{\left(1.5*t_{total}\right)}$$

Running the evolutionary algorithm to optimize the parameters of the model took a significant amount of processing time. In addition, the presence of local optima in the evaluation space made the search for good solutions difficult. We therefore made use of a dedicated server with eight separate cores, and ran six instances of the program in parallel in order to increase the chances of success. Typically, it took several days for an evolutionary algorithm to converge on solutions that were of potential interest to this study. We then had to test them, to analyze the results, and to further fine-tune the parameters of the program before starting the next evolutionary run. It took several months of different evolutionary runs before we finally found the setup and solution described in this paper. Although other solutions probably exist, the current solution was sufficient for our requirements and so we did not make an exhaustive survey.
